# The Dynamic Model of Health Assets: a model development

**DOI:** 10.1177/17579759241248624

**Published:** 2024-06-01

**Authors:** Yuliya Bodryzlova, Gregory Moullec, Michael P. Kelly

**Affiliations:** 1 École de santé publique, Université de Montréal, Canada; 2 Centre de recherche du CIUSSS du Nord-de-l’ Île-de-Montréal, Montréal, Canada; 3 Department of Public Health and Primary Care, University of Cambridge, UK

**Keywords:** positive health, health assets, salutogenesis, health promotion, resistance, resilience, individual traits, environments, social support, community participation, activities, welfare, family and friends, epidemiology

## Abstract

**Aim:**

Epidemiological research on resistance and resilience can build on models of health developed in health promotion. Nevertheless, these models need to be adjusted to approaches currently employed in epidemiology; namely, included concepts should be easy to operationalize, and links between them should be simple enough to enable statistical modeling. In addition, these models should include both individual and environmental assets. The objective of this study is to consolidate the current knowledge on health assets, adjust them to epidemiological research needs, and propose a new model of health assets for epidemiological studies on health.

**Design:**

The conceptual paper was conducted according to the guidelines for the model development.

**Methods:**

The development of the new model was made from the perspective of salutogenesis – the branch of health promotion studying the origins of health. The analysis of literature on health promotion, public health, and positive psychology was conducted to find the links connecting individual and environmental assets.

**Results:**

The newly developed Dynamic Model of Health Assets circularly links individual characteristics, actions, environments, and support. Each preceding component of the model contributes to the following one; each component also independently contributes to resistance and resilience. The new model may guide large-scale epidemiological research on resistance and resilience. The model’s components are easy to operationalize; the model allows for constructing multilevel models and accounting for the dynamic nature of the relationships between components. It is also generic enough to be adjusted to studying contributors to resistance and resilience to different specific diseases.

**Conclusion:**

The new model can guide epidemiological studies on resistance and resilience.

## Introduction

Recently, biomedical science has shown interest in researching protective factors for specific diseases and certain population groups (1–3). Health promotion can add to this with its knowledge of the origins of health (4,5). However, a gap in approaches and perspectives may hinder traditional biomedical science from integrating the body of knowledge developed by health promotion into research practice (6). Health promotion has tended to place great value on qualitative research (7,8) and values a holistic approach to health (9,10). In contrast, biomedical science tends to rely on large-scale quantitative epidemiological studies and tackles relatively narrowly defined conditions as study outcomes (6). In addition, health promotion may operate with complex concepts and consider complex relationships between them, while epidemiological study works with clearly defined variables and may have difficulties operationalizing multicomponent, multilevel complexity (11). Thus, to enhance biomedical research on health with health promotion knowledge of the origin of health, one needs to ‘translate’ some health promotion approaches into epidemiological tools.

In an epidemiological study on health (in contrast to a study on the disease), challenges may arise regarding both its outcome and predictor(s). First, it is not recommended that studies on protective factors use the disease as an outcome, given that risks contribute to disease and protective factors contribute to health reserves (12). Instead, in these studies, a reserve in capacities (functional, anatomical, emotional, etc.) may be an outcome (12). Current knowledge on reserves is limited; in addition, not all reserves are ‘buildable’ and can be a target of prevention strategies. Nevertheless, examples of buildable, specific, and measurable reserves are the cognitive reserve regarding the development of dementia, pre-menopause bone density for osteoporosis in older women, cardiovascular/respiratory/muscular fitness, et cetera (3). To maintain consistency with health promotion terminology, we will further call these reserves ‘positive health’ (12).

Second, challenges may arise with predictors (protective factors). We will refer to the protective factors as ‘health assets’ to maintain consistency with health promotion vocabulary (13–15). The first challenge is a choice of assets to test in relation to a health reserve. They may be less or more specific for specific reserves. For example, reading may offer more significant protection against dementia than physical exercise (16); still, it is less relevant for protecting against cardiovascular disease, where physical activity is more important (17). In addition, the chosen assets must be operationalizable. Ideally, there should be validated tools to measure them. Finally, the set of selected health assets as a whole should be theoretically and logically sound, clear, concise, and comprehensive. In this regard, biomedical studies can draw upon health models commonly used in health promotion.

Choosing a model of health assets as a framework for the study of health may be the most challenging part of a biomedical study about health. Health assets (e.g. individual characteristics, practices, values and attitudes, social networks and community participation, health policies and welfare) are in complex and dynamic interaction. Not relying on health promotion models may lead to omitting important health-promoting factors (e.g. testing cardiovascular fitness in relationships with education without considering the walkability of the neighborhoods where participants live). These omissions may lead to missing opportunities for preventive interventions and policies (e.g. not improving walkability in neighborhoods populated by people with low educational levels). Briefly, in studying health, biomedical science should rely on models developed by health promotion to avoid ‘reinventing wheels, both conceptual and empirical, such that science fails to be incremental and cumulative (18, p.155).’

To our best knowledge, there is little evidence of the application of the scope and utility of health promotion for epidemiological study. As such, the objective of this paper is to review the current state of knowledge on health assets and to develop a model for large-scale epidemiological studies on its basis.

## Methods

We report the model development according to the Jaakkola (19) guidelines for designing a conceptual paper. According to the guidelines, the analytical part of the conceptual paper on the model development should include the presentation of a ‘domain theory’, ‘focal concept’, sub-ordinary concepts and connections between them, and the argumentation part. The domain theory is a system of commonly accepted beliefs and postulates of the field; it is not questioned and provides the ‘Cartesian coordinates’ for the following discussion. The focal concept is a specific problem within the domain theory, which the model under development is trying to explain. The sub-ordinary concepts are the ‘tools’ of the domain theory; they are linked to the practice and are the object of continuous improvement. These concepts and the connections between them constitute (or form) a newly developed model. The utility and coherence of the model is discussed in an argumentation part. For the model development, it should present the ‘explanation and predicting relationships between constructs or identifying novel connections between constructs with the development of theoretical propositions that introduce new constructs and/or relationships between constructs (19, p.22).’ The guidelines propose a *claim-ground-warrant* approach in demonstrating the connections between concepts, where ‘claim’ refers to the thesis to accept, ‘ground’ refers to the reasoning behind, and ‘warrant’ refers to the domain-theory-based underlying assumptions linking grounds to claims.

### Domain theory and focal concept

The domain theory of studies on health is salutogenesis, proposed by Antonovsky in the early 1980s (4,5). According to the original writings on salutogenesis, health and disease have different origins. Studying the origins of health/acting on them alongside the risk factors will enhance public health capacities to improve population health. Beyond this fundamental postulate, which has never been (to our best knowledge) questioned, the original writings suggest that Sense of Coherence (SOC; perception of the world as meaningful, manageable, and comprehensive) is the main contributor to health. It buffers the noxious effect of stress on health. The SOC has its determinants (the General resistance resources). Unlike the fundamental postulate of salutogenesis, the role of SOC, its resources, and possible outcomes of a salutogenic process have been a topic of lively discussion (20–22).

The focal concept of the model is positive health as reserve in capacities, presented earlier in the second paragraph of the Introduction section.

### Sub-ordinary concept 1: Individual characteristics as health assets

The SOC is the most studied contributor to health in studies made from the salutogenesis perspective (4,5). However, other characteristics are proposed for this role as well. Huber *et al*. (23,24) discuss the individual ‘capability to adapt’ as a primary health-related resource. Other authors propose a combination of social competence, resistance skills, commitment to learning, positive values, self-esteem, and a sense of purpose as such (13). Labonté (25) introduces a model encompassing physical, social, and mental dimensions of health through traits like vital energy, community connections, and a sense of purpose. Sen stresses that capabilities – a person’s capacity to achieve a valuable state of being – also contribute to well-being (26). He argued that being included and valued among the capabilities is a basic one. Other authors, predominantly from psychology, also contributed to developing the topic. For example, Ryff *et al*. (27) argue that the experience of well-being is a crucial contributor to health. Vanderburgh states (28) that combining a certain level of individual development with mastery (the ability to manage behavior to achieve significant goals) and a personal approach (orientation to personal development) are sources of health. Jahoda (29) states that a stable self and adequate and stable self-esteem are the key components of positive [mental] health.

### Sub-ordinary concept 2: Environments as health assets

The European Community Health Promotion Indicator Development Model (30) illustrates how social, ecological, and economic resources influence individual health determinants. The ecological (31) positive health model, repeating the ecological risk model (32), places the individual at the center of multiple concentric ecological influences (family, neighborhood, entire society). Being exposed to these interacting influences enables an individual to accumulate positive health. Hancock *et al*. (33) argue that a salutogenic environment comprises friendly, viable environments and prosperous economies. These three factors create equitable, sustainable, and livable communities. Mazzi’s five-factor general resources model views environmental assets as enabling actions, confidence, contact with nature, relaxation, and security (34). Mana and colleagues’ (35) national sense of coherence model emphasizes that societies should be meaningful, manageable, and comprehensible to encourage the maintenance and sustenance of health among its members. Finlay *et al*. (36) stress the importance of social environments equally, such as the state of inclusion of some particular groups (e.g. sexual, religious, or ethnic minorities; poor people; housewives) in local community activities and governance.

### Problematic

Any of these models add to the understanding of health and its origins. However, using only one type of predictor (e.g. individual characteristics) is appropriate when it is known that differences in another type (e.g. environments) are negligible and do not affect the study’s validity. Thus, there may be a need to consider both types of asset, especially at the early exploratory stage of studying the association between assets and positive health. The models, including individual and environmental features, may best serve the epidemiological studies at this stage of evidence development on resistance and resilience.

## Results: Model development

### Reciprocal determinism

Bandura (37) speculated that ‘within reciprocal determinism’ of ‘cognitive, behavioral and environmental determinants [. . .] lies the opportunity for people to influence their destiny. [. . .] Both people and their environments are reciprocal determinants for each other.’ This circular form is present in some other models of health assets, including individual and environmental factors (38–40). However, merely establishing a circular connection between the two is insufficient to create a comprehensive model. It is crucial to explain the interconnections between them.

### From individual characteristics to environments

According to Antonovsky (4,5), perceiving the world as meaningful, manageable, and comprehensive enables good stress-caused tension management and, thus, maintaining health. However, the perception may be biased and prevent a person from successfully resolving complex situations. Inversely, if the perception is correct and the world is meaningful, comprehensive, and manageable – the key word is ‘manageable’ – some actions are needed to overcome a stressful situation. Therefore, not the perception by itself, but timely and well-targeted actions manifest individual characteristics that may contribute to better health. Other writings support this, stressing the role of actions as a link between individual characteristics and good health outcomes. For example, Vanderburgh (28) links individual predisposition and good health to environmental mastery – the capacity to deal with complex environments. Ryff *et al*. (27) argue that the active pursuit of internal, idiosyncratic needs for self-actualization is a crucial contributor to health – even if the quest is accompanied by loss of comfort. Sen (26) discusses the importance of ‘well-being pursuit’ and ‘agency goals’ which go beyond the person’s own life – and emphasizes that the latter ‘weighs’ more for personal wellness. While defining health promotion, Nutbeam and Muscat (41) stress individual and collective actions as a key component of the health promotion process. Finally, Jahoda (29) states that health-creating actions may be of two types: the first type is the actions oriented to self-improvement (‘better self’) and the second type is those oriented to the improvement of environments (‘better world’).

As such, we may link individual characteristics and environments by actions and keep Jahoda’s (29) classification of actions into those oriented to ‘better self’ (e.g. healthy lifestyles or cognitively demanding leisure activities) and ‘better world’ (e.g. community involvement and participation). Individual actions change environments by a critical mass (e.g. exercising in an environment where everyone does). Still, community participation changes environments directly (it might be easier to exercise in an environment where community actions had resulted in a park with sports amenities).

### From environments to individual characteristics

The first writings on the origins of health say little about its social sources and equally pay little attention to health risks embedded in social structures. Antonovsky views the world as a source of stressors and threats (4, pp.76–91): from wars and violence, ‘the unbelievable hell on earth of so large a part of the world’s population (4, p.77),’ to microbiological agents, environmental pollution, radiation hazards, endogenous organisms and so far. (4, pp.76–83) – he hardly mentions the poverty and social inequality as sources of stress by themselves. Even more, he suggests that people at opposite extremities of the social ladder might have the same level of stress due to the relative stability of their social environments. This line of reasoning can be attributed to Antonovsky’s interpretation of stress, primarily focusing on significant traumatic events (‘the difference between a stressor and other types of stimuli [. . .] is a matter of degree (4, p.72)’). The most recent literature, contrarily, shows that the small everyday stress, the ‘wear and tear’ of everyday life, affects health more importantly (42,43). Also, it is shown that the distribution of daily stress follows the social gradient (43,44). People with less access to environmental (economic, cultural, political) resources experience more wear and tear of everyday life and thus enjoy health less. The causes of this phenomenon are also discussed. According to Sen, the lack of freedom to choose a meaningful life due to institutional arrangements constructs an inequality in individual capabilities (45). Marmot responds to Sen’s writing, arguing that the difference in capabilities to control one’s own life and to participate in society makes a difference in health between social standings. He shows on different examples how social hierarchies affect health and calls for improving living standards for the worst-off. Income redistribution by the tax and benefit system is called a solution for inequality in capabilities and, hence, in health. It does not necessarily mean cash transfers, but also investments in public services in education, housing and employment; creation of healthier environments; providing better mobility and transportation, health and social services for people of each age group, gender, and socio-economic group (42, pp.251–252). Furthermore, Hall and Lamont responded to Sen and Marmot by emphasizing the role of cultures in contributing to unequal access to essential resources (46). Given that the economic and culture-based divide between ‘haves and have-nots’ is produced and reproduced by institutions (47), the latter should be reoriented to support and include the deprived members of society (48). Hall and Lamont propose the ‘institutional support’ term, which encompasses policies on welfare, inclusion, and recognition

Institutional support affects individual characteristics: it adds to the complexity of the social world, shaping the human being; it contributes to the adaptability of individuals and the diversity of their life strategies (43,49). As such, we may link environments to individual characteristics by support, informal and institutionalized.

## A final model

**Figure 1.**
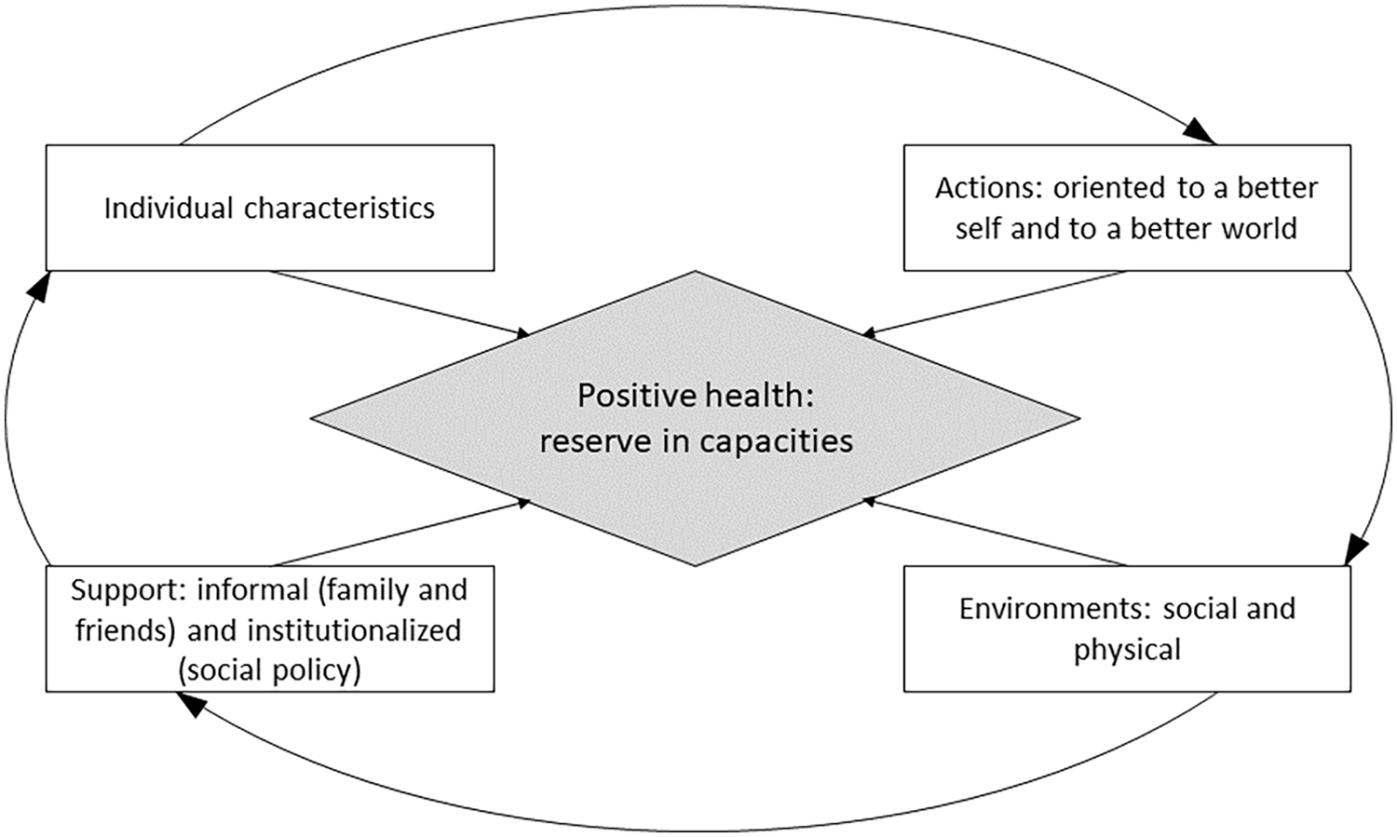
The Dynamic Model of Health Assets.

We integrated all the discussed components into a single model, where each preceding element contributes to the next: some individual characteristics enable actions oriented to a better sense of self or a better world; these actions form physical and social environments, which provide social support: informal or institutionalized. Social support contributes to people’s better adaptability and diversity of life strategies – which results in the reinforcement of individual characteristics building health reserves. In addition, each component contributes to positive health. The graphical representation of the model (the Dynamic Model of Health Assets) is presented in Figure 1.

## Discussion

The model aims to guide epidemiological studies on health assets, informing prevention policies. The latter cannot be constructed exclusively by eliminating immediate health risks at the individual level (smoking, malnutrition, sedentary behavior) but systematically considering larger forces contributing to the adaptation, resistance, and resilience in groups and individuals (50).

Some alternative models are presented in Table 1. Lawrence *et al*. (38) propose a model that examines the interrelationships between human environments, individual and collective activities, and health. The model includes four main domains: policies and programs, the state of green public places, human habitat, and human agency/behavior. Human health and well-being result from the interaction between ecological and individual factors. The human capacity to act (agency) is highlighted as a direct contributor to health and well-being and a force that changes environments. The human agency directly impacts human habitat and green spaces, while programs and policies are shaped both indirectly (through health and well-being and environments) and directly. The model proposed by Hancock *et al*. (33) emphasizes the community actions’ role in promoting health and well-being. The model suggests that effective community governance and education can unite community members, foster community development, facilitate communication, increase participation and empowerment, and uphold civil rights and government accountability. The model of health assets proposed by Rotegård *et al*. (39) highlights the interconnection between internal assets (such as trust, willpower, the strength of motivation, humor, positive thinking, good mood, optimism, hope, courage, will, and goal-directedness) and external assets (such as social support and physical environment). The authors stress that the relationships between internal and external assets are mutually beneficial: external assets can support internal assets and vice versa. Wilson and Cleary’s (51) model of patient outcomes recognizes the interplay between individual and environmental factors in determining health outcomes. The model posits that health production results from symptoms, functions, and health self-perception. Personal factors such as symptom amplification, personality motivation, and values influence health production. Environmental characteristics, such as access to healthcare and informal social support, shape the support provided for heath. Finally, Uexkull and Wsiak’s model, as cited by Lamprecht and Sack (40), highlights the mutual influence between individuals and their surroundings through actions and perceptions. This model can be applied to any level of life organization, from cells to communities.

**Table 1.** Models of health/ health assets including individual and environmental assets.

Author, reference	Model title and concepts	Links from individual characteristics to environments	Links from environments to individual characteristics
Lawrence *et al*. (38)	Positive health effects of the natural outdoor environment in typical populations in different regions of Europe of health and natural environments • Policy, programs • State of public green places • State of human health and well-being • State of human habitat • Human agency • Exposure-based activities	All relationships in the model are bi-directional. • State of public green places is connected to human agency by the exposure-based activities. • Policy, programs connected to human agency • Human agency is connected to the state of human habitat	
Hancock *et al*. (33).	The basic community health indicators model • Community • Environment • Economy • Governance • Education	Governance and education embrace the combination of community, environment and economy	
Rotegård *et al*. (39)	Conceptual model of health assets • Antecedents • Defining attributes ○ internal assets: personality and attitude: relational strength, motivational strength, volitional strength ○ external assets: support, expectations, physical elements • Self-awareness • Mobilization • Consequences	Mutual support	
From Uexkull and Wsiak, cited by Lamprecht and Sack (40)	Bilateral model of health • Receptory sphere • Effectory sphere • Surroundings: ○ problem situation ○ problem solution • Individual: ○ perceiving ○ operating	Actions: operating aspect of the individual proposes the solution to challenging situations by three-step process: assuming its meaning, testing the meaning, and assigning (new) meaning	Perception: surroundings provide individuals with situations perceived as challenges by the individual
Wilson and Cleary (51)	Model of patient outcomes • Biological and physiological variables • Symptom status • Functional status • General health perception • The overall quality of life • Characteristics of the individual ○ symptom amplification ○ personality motivation ○ values, preferences • Characteristics of the environments ○ psychological support ○ social and economic support ○ social and psychological support	The model describes how individual characteristics and environments affect health outcomes: • Symptom amplification and psychological support influence the symptom status • Personality motivation and social and economic support influence the functional status • Values and preferences and social and psychological support influence both general health perception and overall quality of life	
Dynamic Model of Health Assets	• Individual characteristics • Actions ○ ‘better me’ ○ ‘better world’ • Environments ○ physical (e.g. neighborhood design) ○ social (e.g. no discrimination) • Social support ○ informal ○ institutionalized	• Individual characteristics manifest themselves by actions, social support causes the willingness to act	• Environments are formed by individual and collective actions • Environments provide individuals with support needed to act

Theoretically, all of them may serve in epidemiological research on resistance and resilience as they include individual and environmental assets. However, they have some limitations in their scope, content, and ease of operationalization. For example, Lawrence and colleagues’ (38) and Wilson and Cleary’s (51) models are too narrow to explain the entire complexity of the production of health. The Lawrence *et al*. (38) and Rotegård *et al*. (39) models include multiple interactions and feedback loops, which may hamper their operationalization. The mechanisms of mutual influence of environments and individuals are not shown in the models of Hancock *et al*. (33) and Rotegård *et al*. (39). Contrarily, Uexkull and Wsiak’s model (40) is too wide to be operationalized unambiguously.

### Limitations of the new model

First, it does not answer what individual characteristics enable the actions oriented toward a better sense of self and improving environments. Second, the model needs to explain how the support shapes these individual characteristics. Finally, the model omits cultural factors, the importance of which has been widely discussed recently (50,52). Further efforts are needed to incorporate cultural forces in the model of health assets for use in epidemiologic studies.

## Conclusion

The model responds to the main demands to studying resistance and resilience in large epidemiologic studies: first, it enables considering individual and environmental assets; second, it links logically individual and environmental assets by actions and support; and third, its components are easily operationalizable, and there are validated measures for each. Notably, the model considers two features that are rarely considered in epidemiological studies but have repercussions on health: institutionalized support (that comes from social policies on housing, secure employment, access to healthcare, education, etc.) and social environments (a level of inclusion of minority groups in decision making and governance).
